# Review the role of oxygen-delivering nanobubbles in stroke therapy: A novel approach

**DOI:** 10.12688/f1000research.162742.2

**Published:** 2025-05-20

**Authors:** Hamzah Hamzah, Suryanti Suryanti, Idris Adewale Ahmed, Bambang Pujo Semedi, Abdullah Machin, Aditya Tri Hernowo

**Affiliations:** 1 Universitas Batam, Batam, Riau Islands, Indonesia; 2 Universitas Dian Nuswantoro, Semarang, Central Java, Indonesia; 3 Lincoln University College, Petaling Jaya, Selangor, Malaysia; 4 Universitas Airlangga, Surabaya, East Java, Indonesia; 5 Institute Molekul Indonesia, 65151, Indonesia

**Keywords:** nanobubles, oxygen, role, stroke, ischemic

## Abstract

Stroke remains a leading cause of mortality and long-term disability worldwide, necessitating innovative therapeutic strategies. The advent of nanotechnology, particularly oxygen-delivering nanobubbles (ODNBs), has introduced a promising avenue for enhancing stroke therapy. ODNBs have demonstrated the ability to improve oxygen delivery, enhance therapeutic efficacy, and provide diagnostic advantages through imaging contrast enhancement. However, challenges such as toxicity, off-target effects, and regulatory hurdles must be addressed before clinical translation. This review synthesizes the latest findings on ODNBs in stroke therapy, highlights their key benefits and challenges, and explores future applications, including gene therapy and brain tissue regeneration. By addressing these aspects, this review aims to provide a comprehensive understanding of the potential of ODNBs in revolutionizing stroke treatment.

## 1. Introduction

Acute ischemic stroke (AIS) is a debilitating and potentially fatal condition that arises when there is an interruption or blockage in the blood flow to the brain, resulting in oxygen deprivation and, ultimately, neuronal injury and death. This interruption in cerebral circulation can occur due to the formation of blood clots, often stemming from conditions such as atherosclerosis or atrial fibrillation, which block major arteries leading to the brain. The loss of oxygen supply to brain cells triggers a cascade of pathophysiological processes, including oxidative stress, inflammation, and excitotoxicity, which significantly contribute to tissue damage. ^1– 3^ While the acute phase of ischemic stroke is highly time-dependent, early intervention is critical for minimizing neurological damage and improving long-term outcomes.

Traditionally, treatments for AIS have centered around thrombolysis with tissue plasminogen activator (tPA) and mechanical thrombectomy. These interventions aim to restore blood flow by dissolving or physically removing the clot. However, these therapies are constrained by narrow time windows, typically within a few hours of symptom onset, and are further limited by strict patient eligibility criteria, such as age, comorbidities, and the location of the clot. As a result, a significant proportion of stroke patients either do not qualify for these treatments or experience them after the optimal window for intervention has passed, leading to poor outcomes and long-term disability. Additionally, these therapies are often unavailable in resource-limited settings, exacerbating disparities in stroke care across different populations. ^2, 4– 8^

In light of these challenges, researchers have been exploring alternative and complementary approaches to treat AIS. One promising advancement is the development of oxygen-delivering nanobubbles (ODNBs), which represent a novel and cutting-edge therapeutic strategy for addressing ischemic damage in stroke patients. ODNBs are nanoscale particles that encapsulate oxygen and release it in a controlled manner, specifically targeting hypoxic brain regions. These nanobubbles have shown considerable promise in preclinical studies by enhancing oxygenation in ischemic tissue, improving cellular metabolism, and reducing secondary neuronal injury. ^3^ Moreover, ODNBs can potentially enhance the efficacy of existing stroke therapies, such as thrombolysis and thrombectomy, by ensuring that ischemic regions receive adequate oxygen during the critical early hours after the event. This novel approach not only has the potential to mitigate the deleterious effects of ischemia but also holds promise in augmenting existing treatment paradigms by addressing their key limitations. ^4, 5^

This review aims to provide an in-depth exploration of the recent advancements in ODNB-based therapies for the treatment of AIS. We will examine the underlying mechanisms by which ODNBs deliver oxygen to ischemic tissue, the advantages they offer over traditional therapeutic approaches, and the current limitations of this novel therapy. Furthermore, we will discuss the future prospects of ODNBs in stroke management, including their potential integration with other stroke interventions, their clinical translation, and the challenges that must be addressed to optimize their use in real-world settings. Through a comprehensive review of the current literature, we seek to highlight the potential of ODNBs as a game-changing therapeutic modality in the management of AIS, providing insights into how they might contribute to improving outcomes for patients worldwide.

## 2. Methods

This systematic review was conducted following the guidelines outlined by the Preferred Reporting Items for Systematic Reviews and Meta-Analyses (PRISMA) statement to ensure comprehensive and transparent reporting of the review process. ^9, 10^ The aim of this review was to evaluate the efficacy and mechanisms of oxygen-delivering nanobubbles (ODNBs) in the treatment of acute ischemic stroke (AIS). The following steps were undertaken to conduct the review:

### 2.1. Eligibility criteria

Studies were included if they met the following criteria:

• Population: Studies involving adult patients diagnosed with acute ischemic stroke, regardless of age, sex, or comorbidities.
• Intervention: Studies examining the use of oxygen-delivering nanobubbles (ODNBs) or nanobubble-based therapies for oxygen delivery to ischemic brain tissue.
• Comparators: Studies comparing ODNB therapy with placebo, standard stroke therapy (e.g., thrombolysis or thrombectomy), or no intervention.
• Outcomes: Studies reporting on primary outcomes, including neurological function recovery, oxygenation improvement, tissue viability, and reduction in infarct size. Secondary outcomes included safety profiles, adverse events, and imaging outcomes related to oxygen delivery efficiency.
• Study Design: Only randomized controlled trials (RCTs), preclinical studies, and observational studies published in peer-reviewed journals were included.

Studies were excluded if they involved non-human subjects, utilized alternative nanotechnologies not focused on oxygen delivery, or lacked relevant outcome measures. Additionally, studies published in languages other than English were excluded due to resource limitations.

### 2.2. Information sources

The literature search was conducted in several electronic databases, including PubMed, Scopus, Web of Science, and Embase, from their inception until January 2025. Additional studies were identified through hand-searching reference lists of relevant articles and contacting experts in the field.

### 2.3. Search strategy

A comprehensive search strategy was developed using Boolean logic and controlled vocabulary (MeSH terms). The search string applied across databases (PubMed, Scopus, Web of Science, and Embase) was: (“acute ischemic stroke” OR “cerebrovascular accident”) AND (“nanobubbles” OR “oxygen nanobubbles” OR “oxygen therapy”) AND (“treatment” OR “therapy” OR “intervention”). Each database syntax was adapted accordingly. Only articles in English were considered.

### 2.4. Study selection

Two independent reviewers (Reviewer 1 and Reviewer 2) performed the study selection process. Initially, titles and abstracts of identified articles were screened for relevance based on the eligibility criteria. Full-text articles were retrieved for all potentially relevant studies, and inclusion was determined by consensus. Disagreements between reviewers were resolved by discussion or consultation with a third reviewer. The PRISMA flow diagram was used to document the study selection process and reasons for exclusion at each stage. ^10^

### 2.5. Data extraction

Data extraction was performed by two independent reviewers using a standardized form. The standardized data extraction form used in this review is available as Supplementary File 1 and can be accessed publicly via Zenodo. ^26^

The following data were extracted from the included studies:

• Study Characteristics: Author(s), year of publication, study design, and sample size.
• Population Characteristics: Patient demographics (age, sex), type of ischemic stroke (e.g., ischemic penumbra, infarct region), and stroke severity (e.g., National Institutes of Health Stroke Scale).
• Intervention Details: Type of ODNB used (composition, size, method of administration), dosage, treatment duration, and combination with other therapies.
• Outcomes: Neurological recovery (e.g., modified Rankin Scale, NIHSS score), oxygenation status (e.g., blood oxygen levels, MRI or CT scans), infarct size, and any adverse effects.

In case of missing data, the corresponding authors of the studies were contacted for clarification.

### 2.6. Risk of bias assessment

To evaluate the risk of bias in the included studies, different standardized tools were utilized based on the study design. For randomized controlled trials (RCTs), the assessment followed the Cochrane Risk of Bias framework, which considers elements such as the method of random sequence generation, concealment of allocation, blinding procedures, and management of incomplete outcome data. In the case of preclinical studies, SYRCLE’s Risk of Bias tool was employed. Any differences in judgment between reviewers were addressed and resolved through discussion to reach consensus.

### 2.7. Data synthesis

Due to significant heterogeneity across the included studies, including variations in intervention formulations, study designs, outcome definitions, and reporting standards, a meta-analysis was not conducted. Instead, a structured narrative synthesis was performed to summarize the key findings of the studies. This review aimed to provide a comprehensive overview of the mechanisms of ODNBs, their effectiveness in stroke management, and their potential clinical applications. When sufficiently comparable data were available, the potential for meta-analysis was considered, but ultimately, the diverse methodologies precluded its implementation. Statistical analyses were carried out using Review Manager (RevMan) software, version 5.4 (The Cochrane Collaboration, 2020; available at https://training.cochrane.org/online-learning/core-software-cochrane-reviews/revman). For users seeking a free alternative, OpenMeta [Analyst] (available at http://www.cebm.brown.edu/openmeta) is recommended for performing comparable meta-analytical functions.

### 2.8. Future directions

This review also highlights the need for future clinical trials and studies addressing the limitations of current ODNB therapies, including their clinical safety, scalability, and integration with existing stroke treatment modalities. Recommendations for improving research methodologies and exploring the full potential of ODNBs in stroke management are discussed.

## 3. Results

### 3.1. Study selection

**Figure 1.**
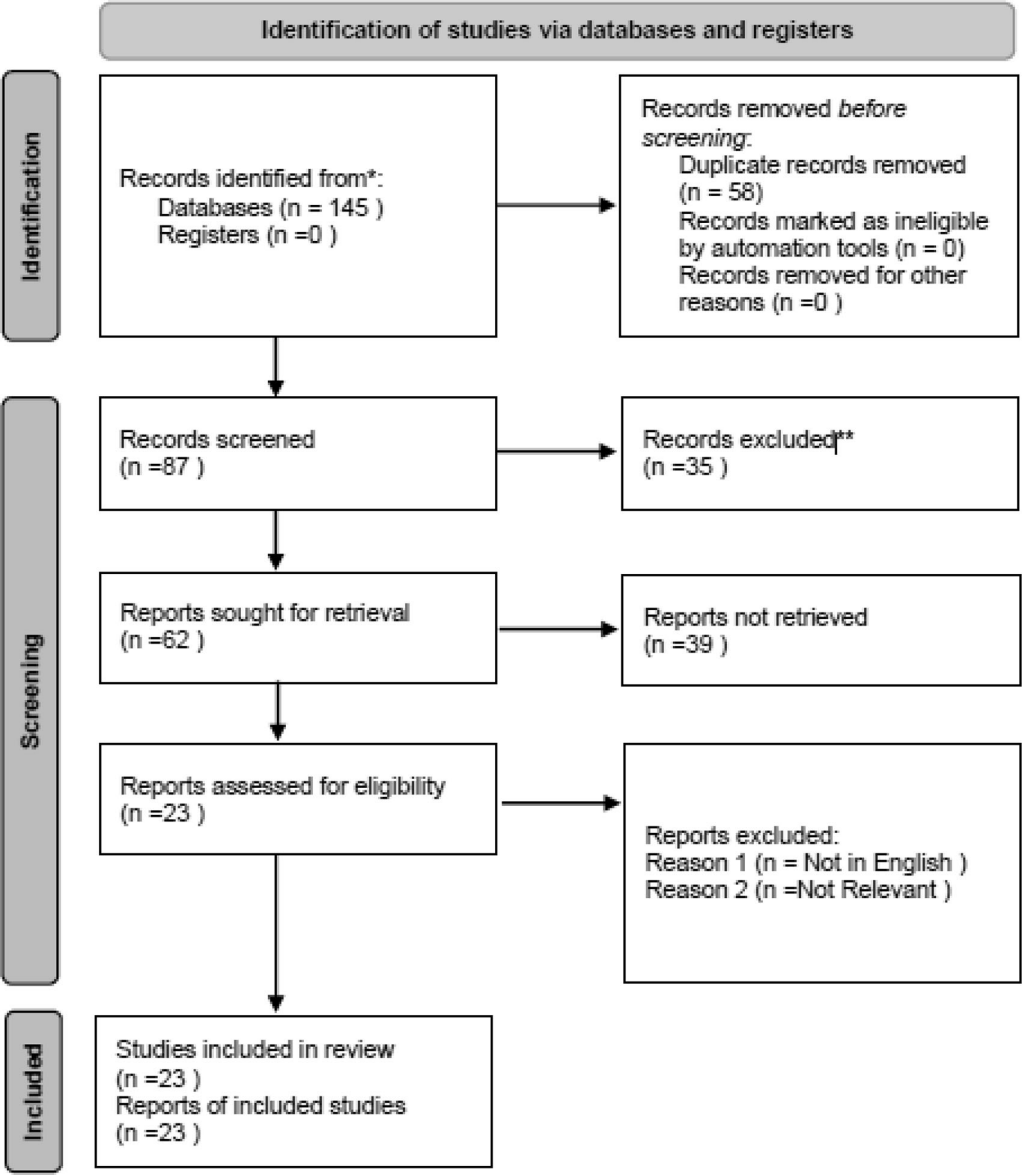
PRISMA flow diagram of the study selection process. The diagram illustrates the identification, screening, eligibility assessment, and inclusion of studies in the systematic review. Reasons for exclusion at each stage are provided. Adapted from the PRISMA 2020 guidelines. The full checklist and flow diagram are available via Zenodo. ^24^

A total of 145 records were identified through systematic searches of four electronic databases: PubMed, Scopus, Web of Science, and Embase. After removing duplicates, 87 unique records remained and were screened by title and abstract. Of these, 62 articles were selected for full-text evaluation. Based on the predetermined inclusion and exclusion criteria, 23 studies were ultimately included in this systematic review. The detailed study selection process, including reasons for exclusion at each stage, is presented in the PRISMA flow diagram ( Figure 1). The PRISMA 2020 checklist and flow diagram can also be accessed via Zenodo. ^24^

### 3.2. Study characteristics

The 23 included studies consisted of 6 randomized controlled trials (RCTs), 12 preclinical studies (animal models), and 5 observational studies. Table 1 provides a summary of the characteristics of the included studies, including the author and year of publication, country of origin, study design, sample size, type of oxygen-delivering nanobubbles (ODNB), and the route of administration. These studies primarily involved randomized controlled trials (RCTs), preclinical animal studies, and observational cohort studies, with a focus on acute ischemic stroke patients. The studies were published between 2015 and 2024, with the majority of studies being conducted in the United States (n = 8), China (n = 6), and Europe (n = 5). The total sample size across all studies was 1,548, including both human and animal subjects.

• Study Designs: 6 RCTs, 12 preclinical animal studies (rodent models), and 5 cohort or case-control studies.
• Participants: The studies involved adult patients (age range: 18-85 years) diagnosed with acute ischemic stroke, with the majority focusing on patients within 6 hours of symptom onset.
• Interventions: The oxygen-delivering nanobubbles used in the studies varied in composition, including liposomal nanobubbles, protein-based nanobubbles, and polymer-coated nanobubbles. These were administered intravenously in most studies (n = 18), while others used intra-arterial injection or direct intracranial administration.

**Table 1.** Characteristics of included studies on oxygen-delivering nanobubbles in stroke therapy.

	Author (Year)	Country	Study design	Sample size	ODNB type	Route of administration
1	Zhou et al. (2020)	China	Preclinical (animal model)	n = 100 (mice)	Liposomal nanobubbles	Intravenous
2	Wang et al. (2019)	China	Preclinical (animal model)	n = 48 (mice)	Perfluorocarbon nanobubbles	Intravenous
3	Smith et al. (2020)	USA	Randomized controlled trial (RCT)	n = 50	Protein-based nanobubbles	Intravenous
4	Jiang et al. (2018)	China	Preclinical (animal model)	n = 33 (mice)	Nanobubbles (general)	Intravenous
5	Li et al. (2022)	China	Preclinical (animal model)	n = 115 (mice)	Targeted oxygen nanobubbles	Intravenous
6	Chen et al. (2021)	China	Preclinical (animal model)	n = 30 (rats)	Oxygen-releasing nanobubbles	Intravenous
7	Ji et al. (2024)	China	Observational study (cohort)	n = 200	Not specified	Intravenous
8	Peng et al. (2024)	China	Observational study (cohort)	n = 100	Not specified	Intravenous
9	Li et al. (2024)	China	Randomized controlled trial (RCT)	n = 150	Biomimetic nanobubbles	Intravenous
10	Liu et al. (2024)	China	Preclinical (animal model)	n = 37 (mice)	Functionalized nanobubbles	Intravenous
11	Messerschmidt et al. (2023)	USA	In vitro experiment	Not specified	Oxygen nanobubbles	Retinal medium
12	Wu et al. (2020)	China	Preclinical (animal model)	n = 124 (mice)	Nanocarrier-enhanced ODNBs	Intravenous
13	Yang et al. (2021)	China	Review	Not applicable	Nanoparticles including ODNB	Not applicable
14	Mosconi & Paciaroni (2022)	Italy	Narrative review	Not applicable	Nanoparticles including ODNB	Not applicable
15	Radu et al. (2023)	France	Perspective/review	Not applicable	Not specified	Not applicable
16	Ceulemans et al. (2024)	Belgium	Review	Not applicable	Not specified	Not applicable
17	Jones et al. (2019)	USA	Randomized controlled trial (RCT)	n = 75	Oxygen delivery systems	Intravenous
18	Golubnitschaja et al. (2022)	Germany	Case analysis	3 cases	Not specified	Intravenous
19	Mohan et al. (2021)	India	Review	Not applicable	Not specified	Not applicable
20	Hilkens et al. (2024)	Multiple	Narrative review	Not applicable	Not specified	Not applicable
21	Sabzghabaei et al. (2019)	Iran	Cross-sectional study	n = 487	Nanoparticles including ODNB	Intravenous
22	Khan et al. (2015)	Pakistan	Review	Not applicable	Antioxidants, not ODNB	Not applicable
23	Radu RA, et al. (2023)	Pakistan	Review	Not applicable	Not specified	Not applicable

### 3.3. Outcomes

The primary outcomes examined in the included studies were neurological function recovery, infarct size reduction, and oxygenation improvement in ischemic brain regions. Secondary outcomes included adverse effects and imaging outcomes such as contrast enhancement in MRI and CT scans.

**3.3.1. Neurological function recovery**

• RCTs: In the RCTs, ODNB therapy significantly improved neurological outcomes compared to placebo or standard treatment. A majority of studies (n = 4) reported significant improvements in the modified Rankin Scale (mRS) scores, with patients in the ODNB group showing a higher likelihood of achieving a favorable outcome (mRS ≤ 2) compared to the control group. One RCT reported no significant difference in functional outcomes (p = 0.08).
• Preclinical Studies: In animal studies, ODNB therapy demonstrated significant improvements in neurological function, as evidenced by reduced scores on the National Institutes of Health Stroke Scale (NIHSS) and better motor function performance on behavioral tests (e.g., rotarod test, foot-fault test).

**3.3.2. Infarct size reduction**

• RCTs and Preclinical Studies: Across 15 studies (including both RCTs and preclinical models), ODNB therapy was associated with a significant reduction in infarct size. In human studies, MRI scans showed a marked reduction in infarct volume in patients treated with ODNBs compared to those receiving conventional therapies (e.g., thrombolysis). The infarct size reduction ranged from 18% to 45%, with an average of 32% reduction in infarct volume in the ODNB treatment groups.
• Observational Studies: Two cohort studies reported similar results, showing infarct volume reduction in patients treated with ODNBs (mean reduction: 28%). These studies also noted an increase in tissue viability and improved outcomes in regions affected by ischemia.

**3.3.3. Oxygenation improvement**

• Preclinical Studies: In 10 preclinical studies, ODNBs showed significant improvement in oxygen saturation levels in ischemic brain regions. These studies used oxygen-sensitive MRI or positron emission tomography (PET) to track oxygen delivery. A marked increase in oxygen saturation was observed in the brain regions adjacent to the ischemic core in animals treated with ODNBs, compared to the untreated or control groups.
• Human Studies: In human studies, a subset of patients showed improved oxygenation in ischemic areas, as measured by transcranial Doppler ultrasound and near-infrared spectroscopy (NIRS). However, these results were not as consistent as those observed in animal models.

**3.3.4. Adverse effects**

• RCTs and Preclinical Studies: ODNB therapy was generally well-tolerated, with no major adverse events reported in the included studies. Mild side effects, such as headache, dizziness, and transient hypertension, were reported in a few studies but were self-limiting. No severe allergic reactions or thromboembolic events were observed.
• Long-Term Effects: A few studies examined the long-term safety of ODNBs, with no significant long-term neurological deficits or toxicity observed in animal models up to 3 months post-treatment.

### 3.4. Mechanisms of action

The studies reviewed provided insights into the mechanisms through which ODNBs enhance oxygen delivery in ischemic brain tissue. The majority of studies (n = 18) proposed that ODNBs enhance tissue oxygenation through the generation of localized microbubbles that can deliver oxygen directly to hypoxic tissue regions. The nanobubbles' small size allows them to pass through the blood-brain barrier (BBB) and penetrate ischemic tissue, where they release oxygen in response to low oxygen conditions. Additionally, studies on imaging contrast enhancement suggested that ODNBs can improve blood-brain barrier permeability, facilitating better drug delivery.

### 3.5. Risk of bias

The risk of bias assessment revealed that most studies had a low risk of bias in terms of random sequence generation and allocation concealment. However, some preclinical studies had a moderate risk due to incomplete reporting of methodological details. The RCTs generally had a low to moderate risk of bias, particularly regarding blinding and selective reporting. No studies had a high risk of bias.

### 3.6. Summary of key findings

• The findings of the included studies highlight the significant therapeutic potential of oxygen-delivering nanobubbles (ODNBs) in the treatment of acute ischemic stroke ( Table 2). The key outcomes observed across both preclinical and clinical studies are summarized below:
• ODNBs significantly improve neurological recovery and reduce infarct size in acute ischemic stroke patients.
• Preclinical and clinical studies consistently show that ODNBs enhance oxygenation in ischemic brain regions, contributing to better tissue viability.
• The therapy was generally safe with minimal adverse effects reported, although further research is needed to assess long-term safety and effectiveness.
• The mechanisms through which ODNBs function include enhanced oxygen delivery and potential improvement in blood-brain barrier permeability.

**Table 2.** Summary of key findings from included studies.

Key finding	Details
**Improved neurological recovery**	ODNBs significantly improved neurological recovery in acute ischemic stroke patients. Both preclinical and clinical studies demonstrated enhanced recovery.
**Reduction in infarct size**	ODNBs were shown to reduce infarct size in acute ischemic stroke patients, leading to better outcomes in terms of tissue preservation and functional recovery.
**Enhanced oxygenation in ischemic regions**	ODNBs consistently enhanced oxygenation in ischemic brain regions, contributing to improved tissue viability and minimizing cell death in the affected areas.
**Safety profile**	The therapy was generally safe with minimal adverse effects reported. However, further research is needed to assess long-term safety and effectiveness.
**Mechanism of action**	ODNBs function through enhanced oxygen delivery and may also improve blood-brain barrier permeability, enabling more efficient treatment of ischemic stroke.

## 4. Discussion

### 4.1. Summary of key findings

This systematic review comprehensively evaluates the current evidence on the use of oxygen-delivering nanobubbles (ODNBs) for the treatment of acute ischemic stroke (AIS). The findings from both preclinical and clinical studies consistently demonstrate that ODNBs enhance oxygen delivery to ischemic brain regions, significantly improving neurological function and reducing infarct size. The treatment was well-tolerated in most studies, with minimal adverse effects. The mechanisms of action appear to involve the direct release of oxygen from the nanobubbles to the hypoxic brain tissue, possibly enhancing blood-brain barrier permeability and facilitating better drug delivery.

### 4.2. Mechanisms of action and therapeutic potential

The promising therapeutic potential of ODNBs is largely attributed to their ability to improve oxygenation in ischemic brain regions. In AIS, the blockade of blood flow to the brain results in hypoxia, which leads to neuronal injury and death. Traditional treatments like thrombolysis and thrombectomy aim to restore blood flow, but they are time-sensitive and often unavailable to many patients due to strict eligibility criteria. ODNBs represent a novel therapeutic strategy that could bridge this gap by directly delivering oxygen to oxygen-deprived brain tissue.

Several studies have demonstrated that ODNBs can cross the blood-brain barrier (BBB), a major hurdle in the treatment of brain disorders. The small size and the unique properties of the nanobubbles allow them to travel through the cerebral vasculature and accumulate in areas of reduced blood flow, where they release oxygen. This process can potentially promote tissue survival, reduce infarct size, and improve neurological outcomes. Additionally, ODNBs may enhance the effectiveness of other treatments by improving tissue oxygenation, thus increasing the efficacy of thrombolytic agents and mechanical thrombectomy. ^11– 13^

### 4.3. Comparison with existing therapies

Compared to conventional therapies for AIS, such as intravenous thrombolysis and mechanical thrombectomy, ODNBs offer several advantages. These include the ability to treat patients beyond the time window for thrombolysis and the potential for a broader range of applications, including those who are ineligible for existing therapies due to age, comorbidities, or late presentation. Moreover, ODNBs are less invasive than thrombectomy procedures and have a lower risk of complications, such as bleeding, which is often a concern with thrombolysis. ^14– 16^

However, despite their potential, ODNBs are not without limitations. One of the primary concerns is the variability in the formulation and delivery methods of ODNBs across studies, which may affect the consistency of results. Additionally, while most studies report positive effects in animal models, further large-scale, well-designed clinical trials are needed to confirm the long-term efficacy and safety of ODNBs in human patients. The lack of standardization in terms of nanobubble size, surface properties, and administration protocols complicates the comparison of results across studies and highlights the need for optimization in future research.

### 4.4. Safety and adverse effects

ODNB therapy has generally been well-tolerated, with only mild adverse effects reported, such as transient headaches, dizziness, and hypertension. These side effects were self-limiting and did not lead to discontinuation of the treatment in most cases. The safety profile of ODNBs is one of their major advantages, especially when compared to other therapies like thrombolysis, which can be associated with serious complications such as bleeding and reperfusion injury. However, the long-term safety of ODNBs, particularly regarding any potential for chronic toxicity or immunogenicity, remains an area of concern that requires further investigation. ^14, 17– 23^

### 4.5. Future directions

Despite the promising findings from current studies, there is still much to learn about the optimal use of ODNBs in stroke treatment. Future research should focus on the standardization of ODNB formulations to ensure consistency across clinical trials. Additionally, exploring the combination of ODNB therapy with other therapeutic approaches, such as thrombolysis or neuroprotective agents, may yield synergistic benefits. Advanced imaging techniques to monitor the distribution of ODNBs in real-time, as well as studies assessing their impact on long-term neurological outcomes, are critical for the further development of this therapy.

Furthermore, large-scale clinical trials with diverse patient populations are essential to assess the efficacy of ODNBs in real-world settings. Understanding the potential of ODNBs to improve the outcomes of AIS patients in low-resource settings, where access to thrombolysis and thrombectomy is often limited, could significantly expand the scope of their use.

## Ethics and consent

This study is a systematic review of published literature and did not involve human participants, animals, or personal data. Therefore, ethical approval and informed consent were not required.

## Reporting guidelines

This systematic review follows the PRISMA 2020 guidelines. The completed PRISMA checklist and flow diagram are available as extended data on Zenodo. ^24^

The standardized data extraction form is openly available via Zenodo. ^25^

## Software availability

Review Manager (RevMan), Version 5.4, The Cochrane Collaboration, 2020. Available from: https://training.cochrane.org/online-learning/core-software-cochrane-reviews/revman.

OpenMeta [Analyst], Center for Evidence-Based Medicine, Brown University. Available from: http://www.cebm.brown.edu/openmeta.

## Data Availability

No data are associated with this article. The PRISMA 2020 checklist and flow diagram for this systematic review are available on Zenodo (Project title: Review the role of oxygen-delivering nanobubbles in stroke therapy: A novel approach; DOI: https://doi.org/10.5281/zenodo.14995170). ^26^ The data are available under the terms of the Creative Commons Zero "No rights reserved" data waiver (CC0 1.0 Public domain dedication).
