# Supplementary material for: Review the role of oxygen-delivering nanobubbles in stroke therapy: A novel approach
Source: F1000Res. 2025 Aug 19;14:406. Originally published 2025 Apr 7. [Version 3] doi: 10.12688/f1000research.162742.3 (PMC12405841; doi:10.12688/f1000research.162742.3)
Supplement: Peer review history

# Reviewer report: Reviewer response for version 3

**DOI:** 10.5256/f1000research.186612.r406872

**Published:** 2025-09-02

**Authors:** Fang Fang

**Affiliations:** 1 National University of Singapore, Singapore, Singapore

The authors have addressed my previous concerns appropriately and carefully. I recommend the manuscript for indexing.

Are the rationale for, and objectives of, the Systematic Review clearly stated?

Yes

Is the statistical analysis and its interpretation appropriate?

Yes

If this is a Living Systematic Review, is the ‘living’ method appropriate and is the search schedule clearly defined and justified? (‘Living Systematic Review’ or a variation of this term should be included in the title.)

Not applicable

Are sufficient details of the methods and analysis provided to allow replication by others?

Yes

Are the conclusions drawn adequately supported by the results presented in the review?

Partly

Reviewer Expertise:

Nanomedicine, Drug Delivery, Self-Assembly, Disease Theranostics

I confirm that I have read this submission and believe that I have an appropriate level of expertise to confirm that it is of an acceptable scientific standard.

# Reviewer report: Reviewer response for version 2

**DOI:** 10.5256/f1000research.181912.r400623

**Published:** 2025-08-14

**Authors:** Fang Fang

**Affiliations:** 1 National University of Singapore, Singapore, Singapore

In this manuscript, Hamzah *et al.* reviewed the role of oxygen-delivering nanobubbles in stroke therapy. The review is systematic and comprehensive, and the method is rigorous and clearly described. After addressing the following minor issues, it could be considered for indexing.

1. In Table 1, a column should be added to indicate whether the study involves combination with other therapies, specifying the applied therapeutic types. Besides, please confirm whether the corresponding references should be provided.
2. The title is “Review the role of oxygen-delivering nanobubbles in stroke therapy: A novel approach,” whereas the detailed discussion on oxygen-delivering nanobubbles was lacking. A summary of the types of nanobubbles and the agents employed to improve oxygenation should be added in the “4. Discussion” section.
3. The challenges in clinical translation and the potential strategies to overcome them should be discussed.

Are the rationale for, and objectives of, the Systematic Review clearly stated?

Yes

Is the statistical analysis and its interpretation appropriate?

Yes

If this is a Living Systematic Review, is the ‘living’ method appropriate and is the search schedule clearly defined and justified? (‘Living Systematic Review’ or a variation of this term should be included in the title.)

Not applicable

Are sufficient details of the methods and analysis provided to allow replication by others?

Yes

Are the conclusions drawn adequately supported by the results presented in the review?

Partly

Reviewer Expertise:

Nanomedicine, Drug Delivery, Self-Assembly, Disease Theranostics

I confirm that I have read this submission and believe that I have an appropriate level of expertise to confirm that it is of an acceptable scientific standard, however I have significant reservations, as outlined above.

# Reviewer report: Reviewer response for version 2

**DOI:** 10.5256/f1000research.181912.r386117

**Published:** 2025-05-29

**Authors:** Kevin Morris

**Affiliations:** 1 Maharashtra University of Health Sciences (MUHS), Nashik, India

The authors have done a decent job in revising the manuscript and updating from the previous version.

Are the rationale for, and objectives of, the Systematic Review clearly stated?

Yes

Is the statistical analysis and its interpretation appropriate?

Partly

If this is a Living Systematic Review, is the ‘living’ method appropriate and is the search schedule clearly defined and justified? (‘Living Systematic Review’ or a variation of this term should be included in the title.)

No

Are sufficient details of the methods and analysis provided to allow replication by others?

Partly

Are the conclusions drawn adequately supported by the results presented in the review?

Partly

Reviewer Expertise:

Neurology, Nanotechnology, internal medicine

I confirm that I have read this submission and believe that I have an appropriate level of expertise to confirm that it is of an acceptable scientific standard.

# Reviewer report: Reviewer response for version 1

**DOI:** 10.5256/f1000research.178993.r379768

**Published:** 2025-05-10

**Authors:** Kevin Morris

**Affiliations:** 1 Maharashtra University of Health Sciences (MUHS), Nashik, India

The authors have done a decent job on a novel topic. However there are few things that need to be addressed before indexing

 1. Clarify search strategy and add Boolean operators to allow for a more comprehensive search strategy for other researchers. 

 2. Add meta-analysis if possible (even limited),

 3. Provide tables summarizing study characteristics and outcomes to remove any doubts about bias in study selection.

 4. Correct minor language issues.

 5. A STANDARD FORM is mentioned but not provided nor any link to the said form is given to check if what is being claimed is true and correct.

Are the rationale for, and objectives of, the Systematic Review clearly stated?

Yes

Is the statistical analysis and its interpretation appropriate?

Partly

If this is a Living Systematic Review, is the ‘living’ method appropriate and is the search schedule clearly defined and justified? (‘Living Systematic Review’ or a variation of this term should be included in the title.)

No

Are sufficient details of the methods and analysis provided to allow replication by others?

Partly

Are the conclusions drawn adequately supported by the results presented in the review?

Partly

Reviewer Expertise:

Neurology, Nanotechnology, internal medicine

I confirm that I have read this submission and believe that I have an appropriate level of expertise to confirm that it is of an acceptable scientific standard, however I have significant reservations, as outlined above.

# Response

**Published:** 2025-05-10

**Authors:** Suryanti Suryanti

**Affiliations:** Faculty of Medicine, Lincoln University College; Universitas Dian NUswantoro, Semarang, Indonesia

We sincerely thank the reviewer for their valuable comments. The following revisions have been made:

1. The search strategy has been clarified and now includes Boolean operators for better reproducibility.
2. Due to significant heterogeneity, a meta-analysis was not feasible; this has been explained in the methods.
3. Two tables summarizing study characteristics and key outcomes have been added to increase transparency.
4. The manuscript has been reviewed and edited for grammar and clarity.
5. The standardized data extraction form is now included as Supplementary File 1 and referenced in the text.

We believe these changes address the reviewer’s concerns and enhance the scientific value of our work.

 Regards,

 Suryanti Chan

 (corresponding author)
